# Enhanced Cellular Cryopreservation by Biopolymer-Associated Suppression of RhoA/ROCK Signaling Pathway

**DOI:** 10.3390/ma14206056

**Published:** 2021-10-14

**Authors:** Tae Wook Lee, Gyeong Won Lee, Seonyeong An, Keum-Yong Seong, Jong Soo Lee, Seung Yun Yang

**Affiliations:** 1 Department of Biomaterials Science (BK21 Four Program), Life and Industry Convergence Institute, Pusan National University, Miryang 50463, Korea; notea007@naver.com (T.W.L.); 22jungbi@gmail.com (G.W.L.); seonyeong510@gmail.com (S.A.); ky.seong0124@gmail.com (K.-Y.S.); 2 Department of Ophthalmology, College of Medicine, Pusan National University, Busan 49241, Korea; 3 Medical Research Institute, Pusan National University Hospital, Busan 49241, Korea

**Keywords:** cryopreservation, hyaluronic acid, dimethyl sulfoxide (DMSO), RhoA/ROCK signaling pathway, polymeric cryoprotectant

## Abstract

With increasing demands on long-term storage of cells, cryopreservation of cells is gaining more importance in cell-based research and applications. Dimethyl sulfoxide (DMSO) is a commonly used chemical cryoprotectant, providing increased cell survival during the freezing process. However, its use is limited in clinical applications due to its low biocompatibility above cryogenic temperatures. Herein, we present a new approach for reducing the use of DMSO in cryopreservation by using biodegradable hyaluronic acids (HAs). By adding HAs into cryoprotectant media containing a low concentration of DMSO, higher cell viability and cell proliferation rate were observed upon thawing after cryopreservation. The HA-supplemented cryopreservation media did not reduce the size of the ice crystal, which significantly influenced cell viability during cell freezing, but decreased the Ras homolog family member A (*RhoA*)/Rho-associated protein kinase (*ROCK*) signaling pathway related to apoptosis. The cell-interactive cryoprotectants containing HA can be applied to the development of a new cryoprotectant that reduces the adverse effect of DMSO.

## 1. Introduction

The importance of cell cryopreservation is emerging because of the development of various fields that use whole cells, including cell therapy [1,2,3,4], regenerative medicine [5,6,7], stem cell research [8], and organ transplantation [9,10,11]. For these cell-based applications, it is necessary to provide a sufficient number of cells and to ensure their activity and stability. Due to cost-related issues and the reduced cell activity during cell maintenance, cryopreservation of cells has been used for the long-term storage of living cells. Cells retain their biological properties even after freezing, and the functions of cells can also be recovered after being thawed [12].

Cryoprotectants are widely used to prevent freezing damage to biological tissues. Since the use of glycerol as a cryoprotectant was first reported in 1949 [13], various cryoprotectants have been used in cryobiology for the cryopreservation of blood cells and bull sperm. Generally, the cryoprotectant enters the cytoplasm, creating an osmotic gradient, which in turn promotes intracellular water efflux, converts the cytoplasm to an amorphous state, and lowers the temperature at which ice begins to form. It also protects cells from freezing by inducing attenuation of osmotic damage due to electrolyte concentration and stabilization of membranes and intracellular structures [14]. Dimethyl sulfoxide (DMSO) is the most commonly used cryoprotectant for the preservation of cells or tissues [15] because it inhibits the formation of ice crystals, which constitute the major physical stress associated with cell cryopreservation [16]. However, due to the toxicity of DMSO, cryopreservation with DMSO could negatively affect cells and tissues and lead to post-transplant complications [17,18]. Thus, it is necessary to develop new cryoprotectants to reduce the use of DMSO in cryopreservation procedures.

Currently, many research efforts have been devoted to developing a novel cryopreservation method that can replace DMSO [19,20]. Inspired by the survival of organisms living in subzero environments, antifreeze proteins (AFPs), found in polar marine organisms, have received great attention as a cryoprotectant [21,22,23]. AFPs inhibit ice recrystallization or allow ice crystals to form in a unique shape, thereby inhibiting cell damage during freezing. However, since AFP shows variable cryopreservation performance depending on the cell type and AFP concentration, it is difficult to establish a standard protocol [24]. In addition, the synthesis process of AFPs, requiring multiple purification steps, is unfavorable for cost-effective production.

Alternatively, synthetic polymers such as polyvinyl alcohol (PVA) have the advantage of being able to mimic the function of AFPs by designing them to inhibit the formation of ice crystals and the advantage of being produced cheaply in large quantities [25]. Although synthetic, non-biodegradable polymer-based cryoprotectants were effective in reducing the size of ice crystals, their residues could potentially lead to less biocompatibility in vivo environments [26]. Recently, biodegradable polymeric cryoprotectants have gained special interest as cryoprotectants due to their complete enzymatic degradation in vivo and excellent biocompatibility [5,11,27,28,29]. Natural biopolymers can induce the release of various growth factors through cell interaction to maintain tissue regulation and homeostasis. The activity of these growth factors can affect systemic or cellular signaling [30].

Recent research demonstrated enhanced cellular cryopreservation by regulating cell signaling [31,32]. For instance, Tian et al. exhibited an increased cryopreservation effect by controlling the *RhoA*/*ROCK1* signal, which increases apoptosis by regulating phosphorylation of the myosin light chain in apoptotic cell [31,32,33]. However, there is still insufficient evidence that biopolymers can increase the cryopreservation effect by regulating apoptosis through the *RhoA*/*ROCK1* signal pathway.

Among various biopolymers, hyaluronic acid (HA), a ubiquitous component of the extracellular matrix in soft tissues, is a good candidate as a cryoprotectant because of its non-toxicity, biodegradability, and large-scale production [28,34]. The HA used for cryopreservation improved cell viability during cell freezing [35,36,37] and showed increased activity of human spermatozoa after exposure to HA [38]. Additionally, when HA-supplemented cryoprotectant was used in human stem cells, the morphology and adhesion of the cells improved [39]. However, despite extensive evidence suggesting the benefits of HA for cryopreservation, the underlying mechanisms remain unclear.

In this study, we aimed to develop a biodegradable polymeric cryoprotectant for reducing the effect of DMSO on cells during cryopreservation. We chose HA as a biodegradable cryoprotectant and examined its effect on cell cryopreservation. In addition, we investigated the cellular signaling pathways induced by polymeric supplements during cryopreservation to reveal potential mechanisms related to HA.

## 2. Materials and Methods

### 2.1. Materials

Murine NIH-3T3 fibroblasts were purchased from ATCC (Manassas, VA, USA). Dulbecco’s modified Eagle’s medium with high glucose (DMEM, DMEM-HPA), fetal bovine serum (FBS, SH30910.03), and penicillin–streptomycin (SV30010) were purchased from Hyclone (Logan, UT, USA). DMSO (DR1022-500-00) and radioimmuno-precipitation assay (RIPA, RC2002-050-00) buffer was purchased from Biosesang (Sungnam, Korea). PVA (weight average molecular weight (M_w_): 8000 g/mol, 360627), thiazolyl blue tetrazolium bromide (MTT, M5655), protease (P4630), phosphatase inhibitors (P5726), and Ponceau S solution (P7170) were purchased from Sigma-Aldrich (St. Louis, MO, USA). Cryofreezing container (5100-0001) and trypan blue (15250061) were purchased from Thermo Fisher Scientific (Waltham, MA, USA). HelixCriptTM 1st-Strand cDNA Synthesis Kit (CDNA50) was purchased from Nanohelix (Daejeon, Korea). HAs with different M_w_ (5000 g/mol referred as oligo-HA and 100,000 g/mol referred as HA) were purchased from SNvia (Busan, Korea). Sodium dodecyl sulfate-polyacrylamide gel (1610302) and nitrocellulose membranes (1703202) were purchased from Bio-Rad (Hercules, CA, USA). Ez-Western LuMiLa kit (DG-WD100) was purchased from DogenBio (Seoul, Korea). *RhoA* (#2117, 21 kDa), *ROCK1* (#4035, 160 kDa), and GAPDH (#2118, 37 kDa) antibody were purchased from Cell Signaling Technology. Goat anti-rabbit IgG, polyclonal antibody (ADI-SAB-300-J) was purchased from Enzo Life Sciences.

### 2.2. Cell Culture

Murine NIH-3T3 fibroblasts were cultured in a 25 cm^2^ culture dish using DMEM supplemented with 10% FBS and penicillin–streptomycin (100 UI/mL). Cultures were maintained at 37 °C in a sterile incubator (Labogene, Seoul, Korea) at 5% CO_2_ until confluence was reached prior to cryopreservation.

### 2.3. Cryopreservation

To prepare cryopreservation media, polymers (PVA, HA, or oligo-HA) were added to DMSO cryoprotective solutions (DMSO 2.5%, FBS 50%, and DMEM 47.5%) with different polymer concentrations; PVA, HA, and Oligo-HA: 1, 5, 10, and 20 mg/mL or polymer free (control). Murine NIH-3T3 fibroblasts (1 × 10^6^ cells) were diluted at 25 °C with cryoprotective solution alone (control) or supplemented with different concentrations of polymer. The samples were transferred to isopropanol-filled cryofreezing containers, cooled down to −80 °C in a low-temperature freezer, and stored at least for 24 h before evaluation. The samples were thawed in water bath at 37 °C for 30 s. An amount of 1 mL sample was diluted in 9 mL culture medium and centrifuged at 300× *g*. After removing the supernatant and collecting the cell pellet, dead cells were counted using trypan blue solution.

### 2.4. Trypan Blue Exclusion Assay

After thawing the frozen NIH-3T3 cells, they were transferred to culture medium and centrifuged at 350× *g* to collect the cells. Collected cells were stained with 0.4% trypan blue, and viable cells were counted by hemocytometry. Images of stained viable cells were captured using an optical microscope (Nikon, Eclipse TS2, Tokyo, Japan) coupled with digital camera (i-Solution IMT, BC, Canada). Cells not stained with trypan blue were counted as live cells, and stained cells were counted as dead cells.

### 2.5. MTT Assay

The proliferation of NIH-3T3 cells was assessed using the MTT assay. Typically, the culture medium in the 24-well plate was completely replaced with 500 μL of DMEM containing 10% (*v*/*v*) MTT solution, and the plate was placed in an incubator at 37 °C for 1.5 h. Formazan quantification was performed with standard graphs containing 0 to 2 × 10^5^ cells. All the parameters were recorded under similar experimental conditions. Formazan in the cells was dissolved in 200 μL of DMSO. DMSO (100 μL) was transferred into a flat-bottomed 96-well plate, and the fluorescence intensity at 570 nm was measured using a microplate reader (Allsheng, Hangzhou, China).

### 2.6. Splat Assay

The size of the ice crystals was measured using a splat assay [21]. An amount of 10 μL of cryopreservation media containing polymeric supplements was dropped from a height of 1.4 m through a 10 cm diameter tube onto a polished aluminum (Al) block cooled with liquid nitrogen. The ice wafer (~1 cm) formed on the Al block was transferred to a precooled glass slide and then moved to a freezing stage connected to a temperature controller (LTS120; Linkam, Epsom Downs, UK) that had been equilibrated at −20 °C. The stage temperature was increased to −8 °C at a rate of 1 °C/min, and the wafer was then left to anneal for 30 min at −8 °C. The temperature was maintained during ice crystal measurement. The ice crystals were visualized using a microscope (DM2700M, Leica, Wetzlar, German) with crossed polarizing filters.

### 2.7. Quantitative Real-Time PCR Analysis

Comparative Ras homolog family member A (*RhoA*) and Rho-associated protein kinase 1 (*ROCK1*) gene expression was accomplished in five groups of NIH-3T3 cells, and each group was harvested after treatment with the specific polymer for 30 min. For each group of NIH-3T3 cells, total RNA was extracted using Trisure reagent. Reverse transcription reactions were performed using the HelixCript^TM^ 1st-Strand cDNA Synthesis Kit. All water used for qPCR was DNase and RNase-free water. In addition, cDNA was synthesized using the RNase inhibitor included in the HelixCriptTM 1st-Strand cDNA Synthesis Kit. The extracted RNA was evaluated using Nanodrop (Nano-400; Allsheng, Hangzhou, China), and all RNA used in experiments had a purity (A260/A280) value of 1.8 or higher. Real-time polymerase chain reaction (RT-PCR) was performed using the StepOnePlus Real-Time PCR System (Thermo Fisher Scientific). For each real-time qPCR reaction, triplication in three independent trials was performed in a 20 μL system, including 10 μL 2×SYBR Green Realtime PCR master mix (Toyobo Co. LTD, QPK-201), 1 μL of 10 pM reverse primer and forward primer, 100 ng template cDNA, and sterile ddH_2_O. The analysis process of qPCR reacting included 30 s at 95 °C, 40 cycles of 5 s at 95 °C, followed by 30 s at 60 °C, and finally at 60 °C extension for 10 min. All primer sequences were checked with Primer-BLAST, all samples were tested using melting curve analysis, and all experiments contained no-template control without any amplification of specific PCR products. The following mouse *RhoA* and *ROCK1* gene-specific primers were used for real-time amplification: mouse *RhoA*, forward 5′-CCG TCG GTT CTC TCC ATA GC-3′ and reverse 5′-TCT CAG ATG CAA GGC TCA AGG-3′; and mouse *ROCK1*, forward 5′-AAG CTT TTG GCA ATC AGC-3′ and reverse 5′-TTC TGT AAA CTT TCC TGC AAG C-3′. Mouse glyceraldehyde 3-phosphate dehydrogenase (GAPDH) was chosen as the housekeeping gene and amplified with the following primers: forward 5′-CGA CTT CAA CAG CAA CTC CCA CTC TTC C-3′ and reverse 5′-TGG GTG GTC CAG GGT TTC TTA CTC CTT-3′. Samples were run in triplicate, and the relative gene expression was calculated using the comparative threshold cycle and normalized to the expression of GAPDH (ΔCt). Results are expressed as fold-changes relative to the mean. The results of qPCR were analyzed by the software (StepOne software v.2.2.2, Applied Biosystems, Waltham, MA, USA) of StepOnePlus Real-Time PCR System, three independent trials were performed, and the outliers among the triplication results were removed and averaged. The specificity of each gene was confirmed through the melting curve of the qPCR results, the amplification efficiency of qPCR was close to 1, and no-template control was not detected in 40 cycles or less. The threshold value of each gene was applied automatically by the qPCR program. The significance of each result was determined by *t*-test.

### 2.8. Western Blotting

NIH-3T3 cells were treated with each polymer for 30 min, PVA (10 mg/mL), HA (10 mg/mL), and oligo-HA (10 mg/mL). Cells were washed twice with phosphate-buffered saline (PBS) and then lysed in RIPA buffer with protease and phosphatase inhibitors. The cell lysates were centrifuged for 15 min at 4 °C, and the supernatants were used for Western blotting. The cell lysate was loaded onto a sodium dodecyl sulfate-polyacrylamide gel at 10 μg each and lysed by electrophoresis, transferred onto nitrocellulose membranes, and stained with 0.1% Ponceau S solution to ensure equal loading of the samples. After blocking with 5% non-fat milk for 30 min, the membranes were incubated with primary antibodies overnight and were incubated for 2 h at 25 °C with secondary antibody diluted 1:5000, and the bound antibodies were incubated with horseradish peroxidase-conjugated secondary antibodies using the Ez-Western LuMiLa kit. Protein bands were visualized using ChemiDoc (Bio-Rad). To quantify the Western blot results, the relative level of the target proteins was normalized to GAPDH. The band intensities were determined using ImageJ software (NIH, Bethesda, MD, USA).

### 2.9. Statistical Analysis

Results of multiple observations are presented as the mean ± standard deviation (SD). For analysis of multivariate data, group differences were assessed using one-way or two-way analysis of variance (ANOVA), followed by Scheffe’s post hoc test.

## 3. Results and Discussion

### 3.1. Effect of Polymeric Supplements on Cell Cryopreservation

**Figure 1:**
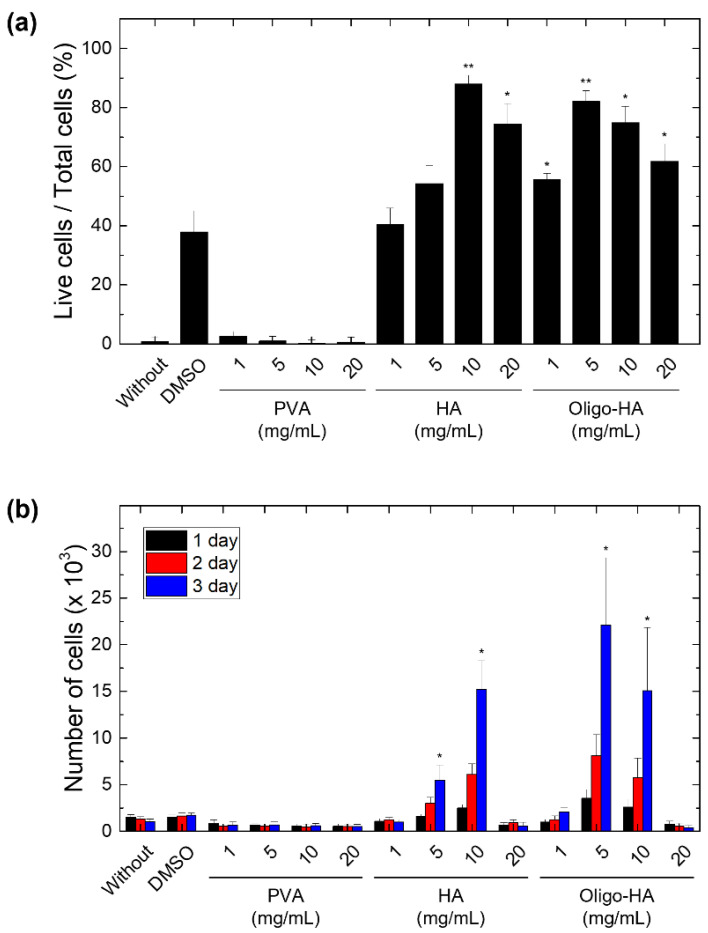
Effect of polymeric supplements on cell cryopreservation. (**a**) The bar graph shows the ratio of living cells per total cells. Polyvinyl alcohol (PVA) and hyaluronic acids (HA) with different concentrations were supplemented to dimethyl sulfoxide (DMSO) cryopreservation media. Dulbecco’s modified Eagle’s medium (DMEM) media without DMSO (without group) was used as a control. (**b**) The graph shows cell proliferation for 3 d after cell thawing. The data represent the mean ± S.D. * *p* < 0.05 and ** *p* < 0.01 vs. DMSO. (n = 5).

To assess the effect of polymeric supplements on cell cryopreservation, cell viability tests were performed using mouse NIH-3T3 cells after freezing in media containing 2.5 wt% DMSO and the tested polymers. To minimize potential side effects, DMSO should be removed before cells are infused into patients. Although DMSO showed concentration-dependent *in vitro* cytotoxicity in the normal culture condition, culture media containing 2.5 wt% DMSO exhibited relatively good cell viability (Figure S1). We used PVA (Mw: 8000 g/mol) and HA (Mw: 100,000 g/mol and 5000 g/mol) as a supplement to DMSO. PVA has been widely used as an effective polymeric cryoprotectant, mainly through its effect of inhibiting ice recrystallization [40]. Due to hydrophilic moieties of the polymer chain, they exhibited good water solubility up to 20 mg/mL. The freezing media supplemented by HA with different molecular weights (referred to as HA and oligo-HA) showed increased viability of NIH-3T3 cells during cryopreservation. Especially, high molecular weight HA at the concentrations of 10 mg/mL showed post-thaw recoveries of up to 90% compared with 37% using DMSO-only cryopreservation (Figure 1a). In contrast, when PVA was added in the cryopreservation media, cell viability was reduced following the cryopreservation of cells (less than 10% post-thaw recovery). As the cryopreservation effect of PVA can be affected by the concentration and molecular weight, the decreased cell viability in PVA-supplemented cryopreservation media might be improved by using ultralow dispersity PVA with proper molecular weight [41]. It was found that 2.5% DMSO cryopreservation media supplemented with HAs showed similar cell viability as the group cryopreserved with standard DMSO cryoprotective solutions (7.5% DMSO) (Figure S2).

To evaluate cell proliferation after cryopreservation, we cultured frozen cells after thawing and measured the growth rate of the cells using the MTT assay. We confirmed that cells treated with HA (10 mg/mL) and oligo-HA (5 or 10 mg/mL) showed normal cell growth after the cryopreservation process. However, the addition of more than 20 mg/mL of HA or oligo-HA to the cryopreservation media inhibited cell growth (Figure 1b). The high concentrations of HA may have induced a cytotoxic effect upon cell freezing by causing damage to the CD44 receptor [42,43,44]. Additionally, we performed cell viability tests using human adipose-derived mesenchymal stem cells and rabbit corneal stromal cells to confirm the cryopreservation effect of HA-supplemented freezing media. Similar to the results obtained with NIH-3T3 cells, it was confirmed that both cells exhibited high cell viability when HA-supplemented freezing media were used (Figure S3).

### 3.2. Ice Recrystallization Inhibition Activity of Polymeric Supplements

**Figure 2:**
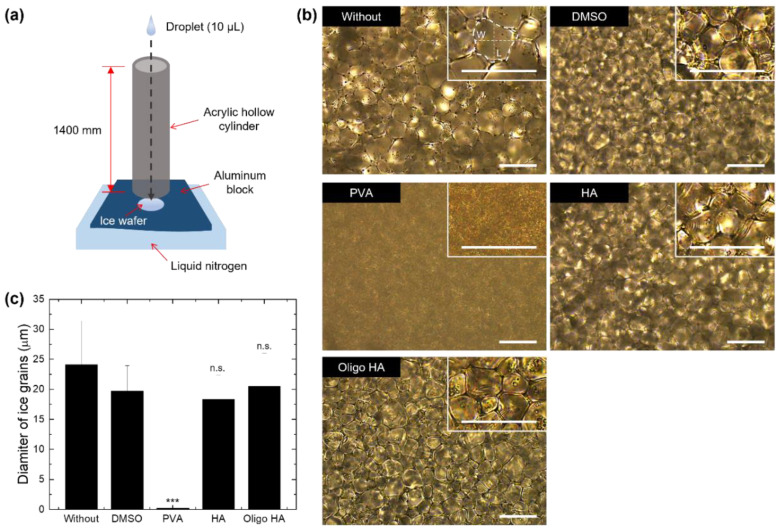
Characterization of ice crystals prepared with cryopreservation media by splat assay. (**a**) Schematic illustration for the experimental set-up of the splat assay. (**b**) Photographs showing the ice grains prepared with different cryopreservation media (frozen DMEM (W/O), with 2.5% DMSO (DMSO) or 10 mg/mL PVA, HA, or oligo-HA). The samples were annealed at −8 °C for 30 min in a splat-cooling chamber. (Scale bar: 50 μm) (**c**) The graph shows the average diameter of ice grains measured from images in Figure 2a. The data represent the mean ± S.D. *** *p* < 0.001 vs. DMSO. n.s. indicates no significance.

Since ice recrystallization can cause mechanical damage during cell cryopreservation, the inhibition of this process is necessary [45,46,47]. To evaluate the ice recrystallization inhibition (IRI) activity of polymeric supplements, we measured the size of ice crystals through a splat assay, illustrated in Figure 2a. A small amount (10 μL) of cryoprotectant-contained solution was dropped onto an aluminum block chilled by liquid nitrogen and annealed at −8 °C for ice crystallization before measuring the size of ice grains using a polarizing microscope [21]. The average diameter was defined as half of (width (W) + length (L)) of an ice grain as shown in the inset of Figure 2b. The PVA-supplemented freezing medium (less than 1 μm) reduced the size of ice crystals compared with the DMSO only group (19.7 ± 4.2 μm) (Figure 2b,c). This IRI activity of PVA is attributed to the weak and reversible adsorption of PVA on the ice surface [21]. In contrast, the freezing media supplemented with HAs (HA and oligo-HA groups) showed similar ice crystal sizes to those of the DMSO group. Given the enhanced cell cryopreservation in HA-supplemented freezing media (Figure 1a), this result suggests that the addition of HA to the freezing media has another function of increasing cellular cryopreservation rather than inhibiting ice formation and growth, which is the working mechanism of common polymeric cryoprotectants such as PVA.

### 3.3. Downregulation of RhoA/ROCK1 following Polymer Treatment

**Figure 3:**
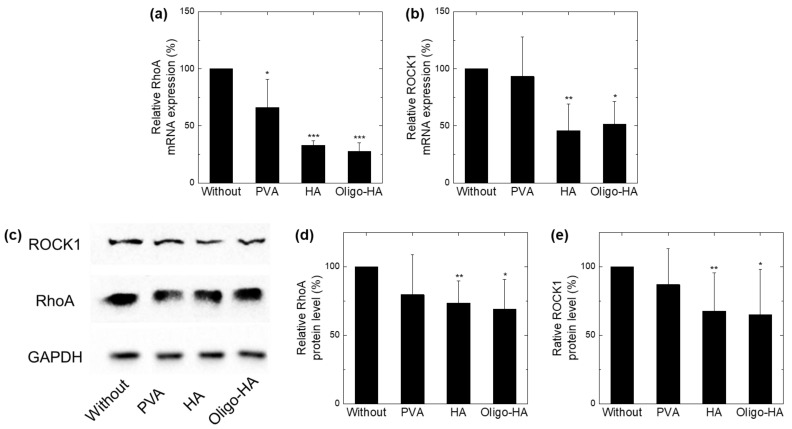
Ras homolog family member A (*RhoA*)/Rho-associated protein kinase (*ROCK*) pathway regulation effect of polymeric supplements. NIH-3T3 fibroblast cells were treated with PVA (10 mg/mL), HA (10 mg/mL), or oligo-HA (10 mg/mL) for 30 min, and expression levels of *RhoA*, *ROCK1*, and glyceraldehyde 3-phosphate dehydrogenase (GAPDH) were determined by quantitative real-time polymerase chain reaction (qRT-PCR) and Western blotting analysis. (**a**,**b**) mRNA levels of (**a**) *RhoA* and (b) *ROCK1* determined through qRT-PCR at 30 min after each treatment with polymers. The mRNA levels were normalized with GAPDH. Data represent mean ± S.D (n = 5). (**c**) Representative images showing Western blotting data from four independent experiments. (**d**,**e**) Bar graphs show Western blot analysis and quantification of (**d**) *RhoA* and (**e**) *ROCK1* protein levels. * *p* < 0.05, ** *p* < 0.01, *** *p* < 0.001 vs. without.

We investigated other mechanisms, apart from that of the ice crystal size reduction, that could increase the effectiveness of cryopreservation. Previous studies have shown that *RhoA* and *ROCK1* signals are important for cell survival during freezing [31,32]. To evaluate whether the polymers modulate the cellular *RhoA/ROCK1* signaling pathway, we investigated the expression of *RhoA* and *ROCK1* in NIH-3T3 cells *via* qRT-PCR and Western blotting. The expression of *RhoA* and *ROCK1* mRNA after treatment with HA or oligo-HA was significantly downregulated in NIH-3T3 cells compared with that in the untreated cells (Figure 3a,b). Similarly, when tested with HA or oligo-HA, Western blot results showed downregulation of total *RhoA* and *ROCK1* protein in NIH-3T3 cells compared with the untreated cells (Figure 3c,d). In contrast, when PVA was treated with NIH-3T3 cells, mRNA and protein expression of *RhoA* and *ROCK1* did not decrease. These results show that HA and oligo-HA decreased the expression of *RhoA*/*ROCK1* in NIH-3T3 cells, implying that the HA-supplemented freezing media contribute to cell cryopreservation through the *RhoA/ROCK* signaling pathway.

In a recent study, signaling induced by interaction between HA and CD44 receptor of cancer cells was shown to increase protein kinase C and tyrosine kinase activity, thereby lowering oxidative metabolism in the cells [37,48]. The complex formed by the HA–CD44 interaction is known to activate cell survival anti-apoptotic proteins, which is similar to our findings in this study [48,49,50,51]. This is probably because HA decreases the *RhoA/ROCK* signaling pathway in cells, thus reducing apoptosis during cryopreservation. These findings provide another perspective for cell cryopreservation using cell-interactive cryoprotectants. In addition, since biopolymers have advantages of high biocompatibility and low cytotoxicity compared with petroleum-based synthetic polymers [27,29], cryopreservation medium supplemented with HA and low concentration of DMSO would be beneficial for clinical applications. Since CD44, a receptor for HA in fibroblasts, could regulate collagen accumulation and fibroblast motility [52,53,54], further studies are needed to investigate the effect of supplemented HAs on cellular functions such as ECM production.

## 4. Conclusions

In this study, we report a simple and effective method for reducing the use of DMSO in cryopreservation by using biodegradable hyaluronic acids (HAs). By adding HAs into cryoprotectant media containing a low concentration of DMSO, higher cell viability and cell proliferation rate were observed upon thawing after cryopreservation. When the HA was added at 1 wt% to the freezing medium compared with DMSO alone, post-thaw recoveries of more than 2-fold were measured. Although HA did not contribute to inhibiting ice recrystallization, it was found that HA was involved in inhibiting *RhoA* and *ROCK* signals, thereby reducing apoptosis during cryopreservation. These findings suggest a new cryopreservation method by using a cell-interactive polymeric cryoprotectant with low concentration of DMSO. Since the safety of HA has been proven in the medical field, cryopreservation medium containing HA would be advantageous for clinical translation. However, since this work was performed during a short freezing period at −80 °C, further studies are needed to confirm the cryopreservation effect of HA on cells after long-term storage in liquid nitrogen.

## Data Availability

The data that support the findings of this study are available from the corresponding author upon reasonable request.

## Supplementary Materials

The following are available online at https://www.mdpi.com/article/10.3390/ma14206056/s1, Figure S1: The effect of DMSO concentrations on cell viability, Figure S2: Cell viability after cryopreservation with different cryopreservation media, Figure S3: Cell viability tests using human adipose-derived mesenchymal stem cells and rabbit corneal stromal cells to confirm the cryopreservation effect of HA.
