# Supplementary material for: Enhanced Cellular Cryopreservation by Biopolymer-Associated Suppression of RhoA/ROCK Signaling Pathway
Source: Materials (Basel). 2021 Oct 14;14(20):6056. doi: 10.3390/ma14206056 (PMC8540109; doi:10.3390/ma14206056)
Supplement: Supplementary file 1 [file materials-14-06056-s001.zip › materials-1386205-supplementary.pdf]

SUPPORTING INFORMATION

Enhanced cellular cryopreservation by biopolymer-associated suppression of RhoA/ROCK signaling pathway

Tae Wook Lee[1*], Gyeong Won Lee[1*], Seon Yeong An[1], Keum-Yong Seong[1], Jong Soo Lee[2#] and Seung Yun Yang[1#]

1. Affiliation 1 ; Department of Biomaterials Science (BK21 Four Program), Life and Industry Conver-gence Institute, Pusan National University, Miryang 50463, Republic of Korea
2. Affiliation 2 ; Department of Ophthalmology, Pusan National University College of Medicine & Medical Research Institute of Pusan National University Hospital, Busan 49241, Republic of Korea

* These authors contributed equally to this work

\# Correspondence: S. Y. Yang (E-mail: syang@pusan.ac.kr), J. S. Lee (E-mail: jongsool@pusan.ac.kr)

KEYWORDS: Cryopreservation; Hyaluronic acid; Dimethyl sulfoxide (DMSO), RhoA/ROCK signaling pathway; Polymeric cryoprotectant

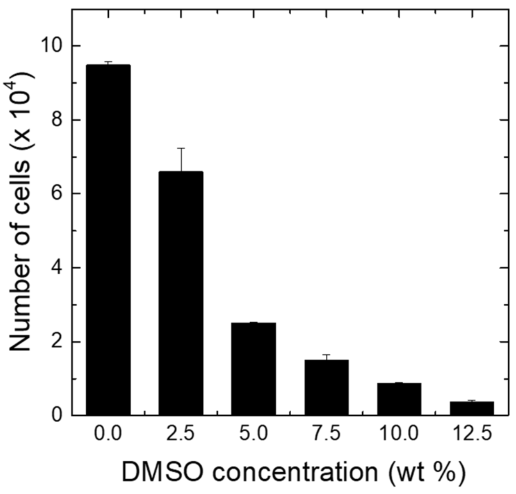

Figure S1. Cell viability tests using NIH-3T3 fibroblasts cultured in media containing different DMSO concentrations for 3 h.

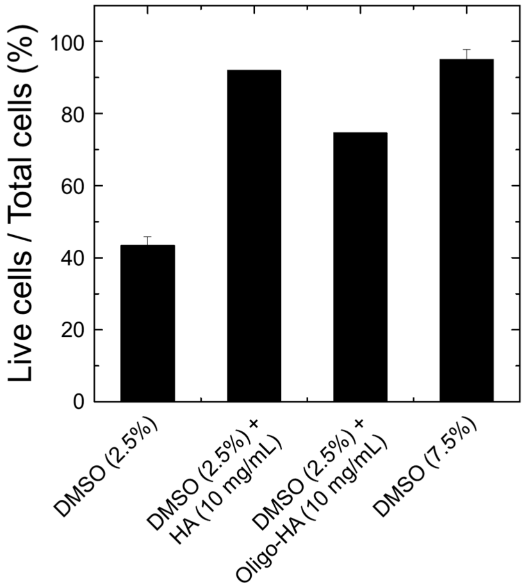

Figure S2. Bar graph showing the percentage of live cells per total cells after cryopreservation with different cryopreservation media.

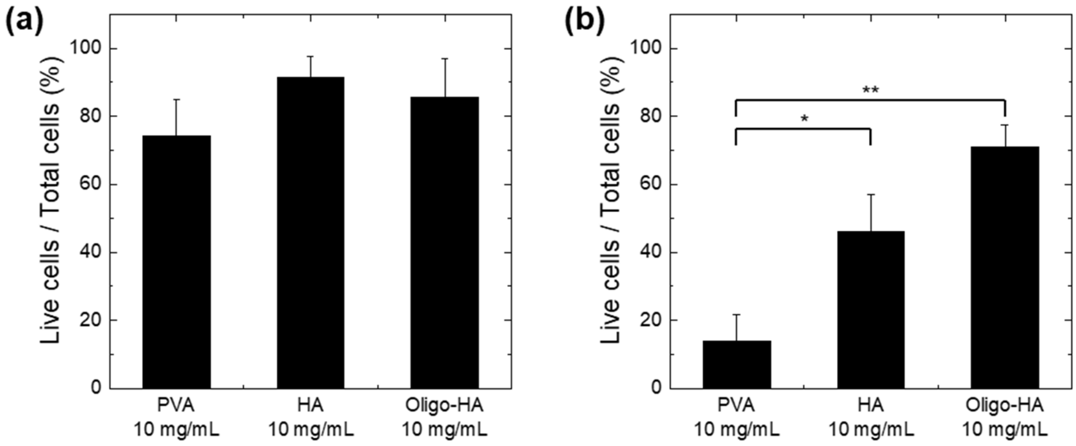

Figure S3. Bar graphs showing the ratio of viable cells per total cells in (**a**) human adipose-derived mesenchymal stem cells and (**b**) rabbit corneal stromal cells measured by the trypan blue exclusion assay. PVA, HA and Oligo-HA with 10 mg/mL concentrations were supplemented to 2.5 w% DMSO cryopreservation media. The data represent the mean ± S.D. * $p < 0.05$ and ** $p < 0.01$.
